# Comparative study of the modified VISTA technique (m-VISTA) versus the coronally advanced flap (CAF) in the treatment of multiple Miller class III/RT2 recessions: a randomized clinical trial

**DOI:** 10.1007/s00784-022-04746-w

**Published:** 2022-10-20

**Authors:** Aitziber Fernández-Jiménez, Ruth Estefanía-Fresco, Ana María García-De-La-Fuente, Xabier Marichalar-Mendia, José Manuel Aguirre-Urizar, Luis Antonio Aguirre-Zorzano

**Affiliations:** 1 Department of Stomatology II, Faculty of Medicine and Nursing, University of the Basque Country/Euskal Herriko Unibertsitatea (UPV/EHU), Barrio Sarriena s/n, 48940 Leioa, Bizkaia Spain; 2 Department of Nursing I, University of the Basque Country/Euskal Herriko Unibertsitatea (UPV/EHU), Bizkaia, Spain

**Keywords:** Gingival Recessions, Plastic surgery, Connective tissue, Clinical trial

## Abstract

**Objectives:**

To compare the percentage of mean root coverage (MRC%) obtained in the treatment of multiple Miller class III/RT2 gingival recessions using the modified VISTA (m-VISTA) technique versus the coronally advanced flap (CAF) technique, using a connective tissue graft (CTG) in both cases.

**Materials and methods:**

Twenty-four patients were randomly treated with m-VISTA (test group (TG) = 12) or CAF (control group (CG) = 12). A calibrated, experienced, and blinded examiner collected data related to multiple periodontal clinical variables, especially the recession (REC) in order to calculate the MRC% at 6 and 12 months, which was the primary outcome of the study. Also, the radiological bone level, the characteristics of the CTG, and postsurgical incidences were assessed. Finally, a descriptive and an analytical statistical analysis of the variables and their associations was performed.

**Results:**

The recessions (*n* = 84) were located mainly in the mandible (*n* = 65) and in posterior sectors (premolars: *n* = 35; molars: *n* = 8). At 6 months, the MRC% was 61% (2 mm) for both study groups, and at 12 months, it increased to 73.26% (2.11 mm) in the TG and decreased to 56.49% (1.78 mm) in the CG.

**Conclusion:**

When approaching multiple Miller class III/RT2 recessions, there were no statistically significant differences in the MRC% at 6 and 12 months between the group treated with the m-VISTA technique and the group treated with the CAF.

**Clinical relevance:**

The characteristics of the m-VISTA technique, such as the closed approach, the mobilization of the papilla, and the coronal stabilization of the CTG, could facilitate the maturation of the tissues in the treatment of Miller class III/RT2 recessions. This would favor better root coverage.

**Trial registration:**

NCT03258996.

**Supplementary Information:**

The online version contains supplementary material available at 10.1007/s00784-022-04746-w.

## Introduction

Gingival recession is defined as the apical displacement of the marginal gingival tissue towards the cementoenamel junction (CEJ) with exposure of the root surface [1, 2]. It is a sign that is frequently associated with the attachment loss caused by periodontitis. When this loss of interproximal support is not greater than the buccal attachment loss, the GR is classified as Miller class III [3] or Cairo RT2 [4].

Until now, there are few studies on multiple Miller class III [3] or Cairo RT2 [4] gingival recessions, limited to case series, retrospective studies, and eleven randomized clinical trials (RCTs) [5–13]. Moreover, only in three of these RCTs [10, 12, 13] were multiple Miller class III [3] gingival recessions treated and followed up for more than 12 months (12 to 36 months) [10, 12, 13]. At 12-month follow-up, the percentage of mean root coverage (MRC%) ranged from 62.83% [10] to 79.10% [12] and the percentage of complete root coverage (CRC%) from 11.51% [12] to 20.47% [13], with the root coverage (RC) values decreasing over time after 12 months [13]. These trials mainly evaluated coronally advanced techniques with the combined use of a connective tissue graft (CTG), assessing if enamel-derived proteins would provide any additional benefits in the treatment of these challenging recessions, not showing any statistically differences between the groups [12, 13].

To the best of the authors’ knowledge, there are no studies that compare the surgical techniques used to prepare the recipient bed in periodontal plastic surgery in this type of recessions. In fact, this need was addressed in the Regeneration Workshop of 2015 [6, 7] as a priority for future research.

In this regard, and although the coronally advanced flap (CAF) [14] has been successfully used for years, new techniques have been suggested to minimize the surgical trauma and increase the vascularization in the recipient site [15], thereby obtaining better clinical results. One of these procedures would be an access tunnel technique using a modified subperiosteal vestibular incision (m-VISTA) [16].

Hence, the aim of this RCT was to evaluate if there were no differences with respect to MRC% in the treatment of Miller class III [3]/RT2 [4] multiple gingival recessions between the CAF [14] and the m-VISTA technique [16], both combined with a CTG.

## Materials and methods

### Study design and population, inclusion, and exclusion criteria

This investigation was designed as a triple-blind RCT with a follow-up of 12 months. The study was registered at clinicaltrials.gov as NCT03258996. Patients were recruited (AFJ) among those attending the Master in Periodontics and Osseointegration at the University of the Basque Country (UPV/EHU) (Spain) between December 2017 and February 2020. During the first visit, potential participants were consecutively screened to determine their eligibility according to the following inclusion and exclusion criteria:

Inclusion criteria:

- Age > 18 years
- Multiple Miller class III [3]/RT2 [4] gingival recessions, defined as the presence of at least three adjacent recessions or three recessions located in the same quadrant with a depth of ≥ 2 mm
- Periodontal plastic surgery indicated for esthetic reasons, recurrent inflammation, progressive recession or dentin hypersensitivity
- Absence of active periodontal pathology
- Full-mouth plaque index (FMPI) [17] and full-mouth bleeding index (FMBI) [18] ≤ 15%.

Exclusion criteria:

- Smokers of ≥ 10 cigarettes/day
- Systemic conditions that contraindicated surgery
- The use of analgesic and/or anti-inflammatory drugs in the last 72 h
- The use of opioid drugs, anticonvulsants and/or antidepressants, except selective serotonin inhibitors, i.e., those drugs that act by reducing the painful experience
- pregnancy or nursing women

This study protocol was approved by the Ethics Committee of the UPV/EHU (M10/2017/042). All patients gave their informed consent, and all study procedures were performed according to the criteria included in the Declaration of Helsinki (1975; revised in 2013). The Consolidated Standards of Reporting (CONSORT) guidelines [19] for clinical trials were followed. Patients signed informed consent regarding publishing their data and photographs.

### Blinding and calibration

The reproducibility of the masked clinical examiner (REF) was assessed by measuring the recessions of four patients who were not included in the study at two different visits that were separated by at least 24 h. The data were used to determine the intraclass correlation coefficient, which was considered acceptable at values of at least 0.75.

In addition, to determine the surgical technique to be used, the patients were randomized in blocks of four using a statistical software (IBM SPSS® Statistics 20.0;IBM, Chicago, IL, USA) [20] (AMGF) and the assignments were kept hidden by a clinical monitor (AMGF) in opaque envelopes until the time of the intervention. The patients, the calibrated clinical examiner (REF), and the biostatistician (XMM) were blinded to the treatment allocation.

### Surgical techniques

**Fig. 1:**
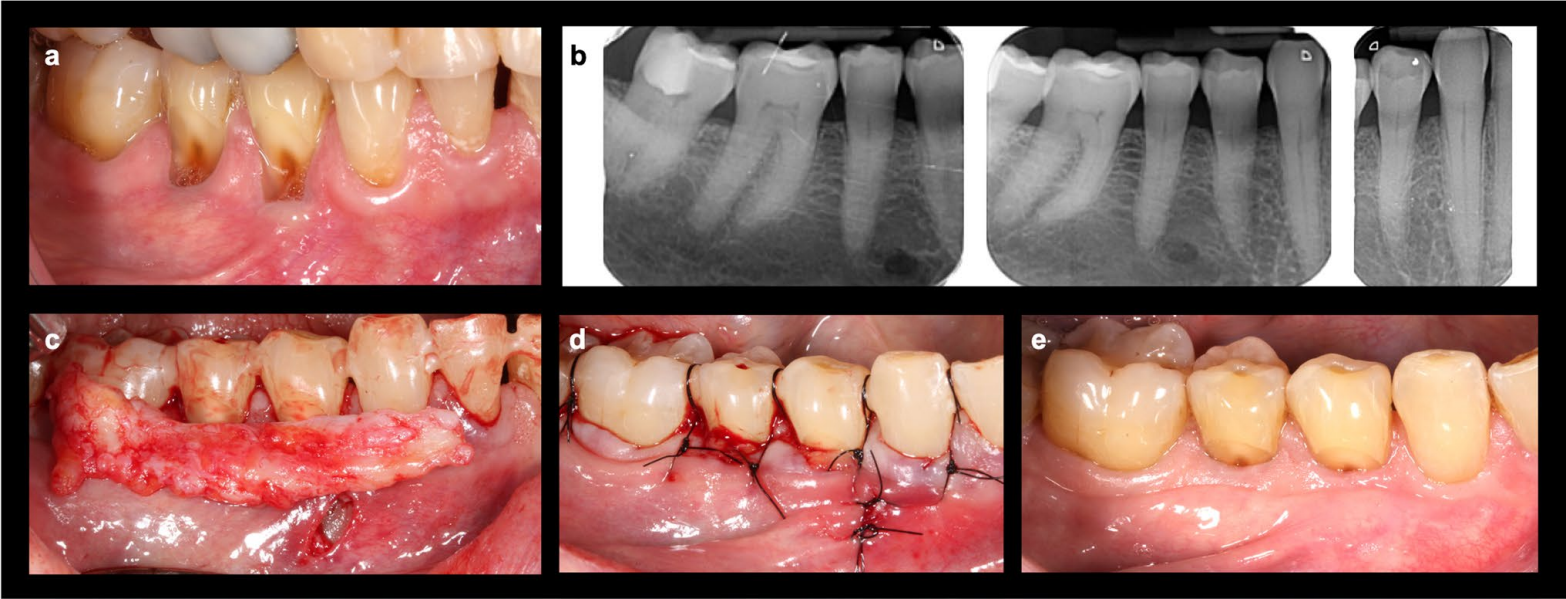
**a** Miller class III/RT2 multiple gingival recessions from 4.3 to 4.6. **b** Interproximal bone loss in the teeth with recessions to be treated. **c** Mucoperiosteal tunnel and elevation of the papillae through a single vestibular incision with a CTG from the palate. **d** Double-crossed vertical sutures (tunnel-papillae-graft) anchored at each contact point and vertical incision closure with single interrupted sutures (m-VISTA). **e** Clinical outcome at 1-year follow-up

**Fig. 2:**
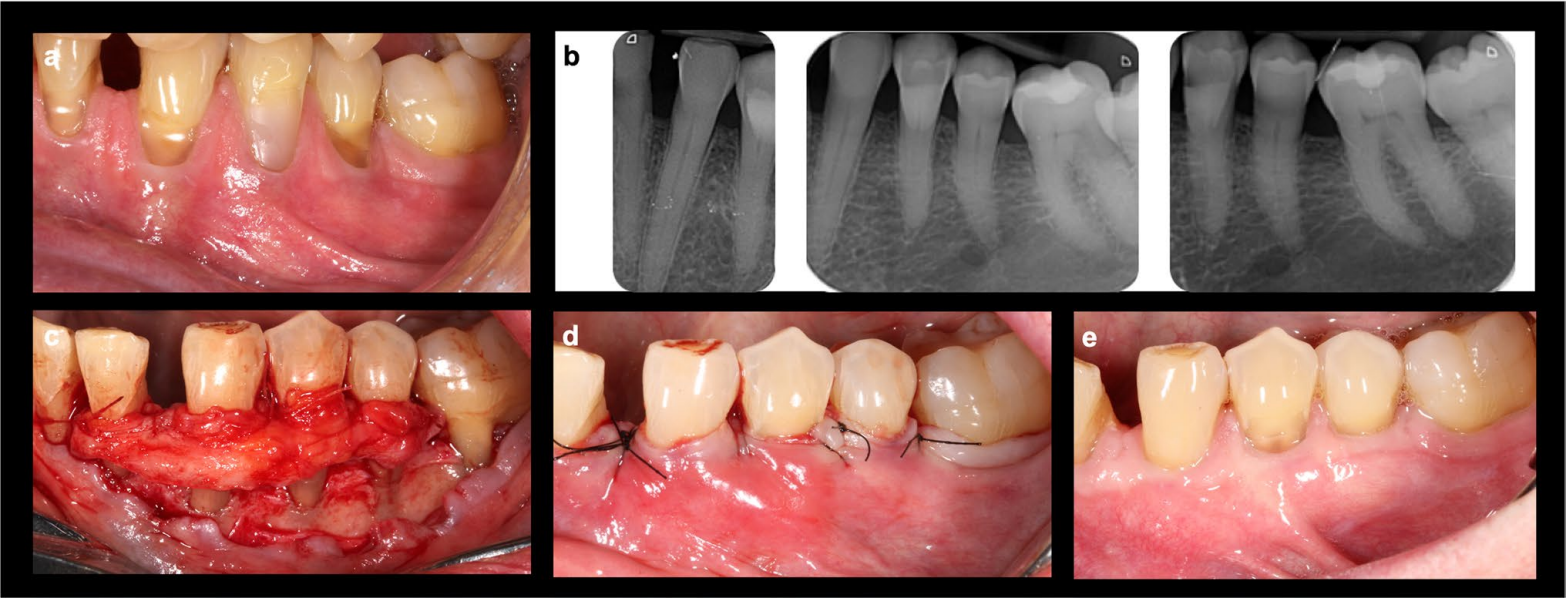
**a** Multiple Miller class III/RT2 gingival recessions from 3.3 to 3.5. **b** Interproximal bone loss in the teeth with recessions to be treated. **c** Split-full-split thickness flap elevation with the CTG secured in position. **d** Coronal stabilization of the flap with sling sutures (CAF). **e** Clinical outcome at 1-year follow-up

The surgical techniques performed for both the test group (TG) (m-VISTA) [16] and the control group (CG) (CAF) [14] are described in detail in the articles by Fernández-Jiménez et al. (2021) [16] (Fig. 1a–e) and Zucchelli & De Sanctis (2000) [14] (Fig. 2a–e), respectively. All the interventions were carried out by training surgeons supervised by an experienced periodontist (AFJ).

Briefly, in the m-VISTA technique [16], a mucoperiosteal tunnel is performed through a single vestibular incision, with the complete elevation of the involved papillae and complete traction of the tunnel-papillae-graft complex using double-crossed vertical sutures [21] onto the contact points previously splinted with composite. In contrast, the CAF technique [14] consists of a flap without releasing incisions extending at least one tooth adjacent to the area to be treated. It begins with oblique submarginal incisions, taking the deepest recession as the point of reference, following with intrasulcular incisions at the recession defects. The flap is then elevated with a split-full-split thickness approach to favor its corono-apical displacement, so that it can be coronally stabilized attaching it to the previously de-epithelialized papillae, by means of periosteal dissection and sling sutures.

In all cases, the CTG was harvested using the UPV/EHU technique [22], which begins with an intrasucular incision along the involved teeth, preserving the interdental papillae, to raise a full thickness flap (FTF) in the palate. Then, the FTF is dissected with a 15C blade by holding it with the tissue forceps, obtaining the CTG. If needed, the length of the graft was extended using the expanded mesh procedure [23] which was described in 2004. The procedure consists of performing alternative incisions on each edge of the graft, to expand it and be able to cover all the teeth with recession (recipient bed) [23]

All patients followed this postsurgical protocol: amoxicillin 875 mg/clavulanic acid 125 mg orally every 8 h for 7 days, ibuprofen 400 mg orally every 8 h for 2 days, a 0.12% chlorhexidine digluconate mouthwash twice a day for 6 weeks, avoiding toothbrushing of the surgical area for 3 weeks, cold applications for 2 days, soft diet and no physical exercise during the first week after the surgery.

Sutures were removed from the palate and the recipient bed at 1 week and 14 days, respectively. Then, patients were instructed to resume oral hygiene, using an ultra-soft toothbrush and the Stillman technique [24] from the third to the sixth week after the surgery, and their regular oral hygiene habits from then on. Finally, all patients were enrolled in a supportive periodontal therapy program 1, 3, and 6 months after the intervention.

### Outcome measures

A single, calibrated, and blinded examiner (REF) recorded all the variables in the UPV/EHU Dental Clinic (Spain).

The location and the number of teeth for intervention were recorded and the following clinical variables were measured in millimeters using a periodontal probe (PCP-11, Hu-Friedy® Mfg. Co. LLC, Chicago, USA): gingival recession (REC), probing depth (PD), clinical attachment level (CAL = REC + PD), and keratinized gingiva width (KGW) were measured in the mid vestibular, gingival recession width (GRW) was measured horizontally at the level of the CEJ, and the distance from the contact point to the interdental papilla (PC-IP) of the tooth with recession was measured both mesially and distally. As non-carious cervical lesions (NCCLs) were not excluded in the present study, when the CEJ was not detectable, it was determined considering the interdental CEJ which was easily identified by elevating the interdental soft tissue with a probe [25]. Periodontal parameters such as REC, PD, and CAL, together with the FMPI [17] and FMBI [18], were collected for all teeth present, except the wisdom teeth. The rest of the parameters were recorded only for the recessions to be treated, for which the initial radiological bone level (RxBL) was also measured using the ImageJ® programme [26] in calibrated radiographs: the distance in millimeters from the CEJ to the first interproximal bone contact was measured in a straight line on the mesial and distal surfaces.

Intraoperatively, the characteristics of the CTG (length, width, and thickness) were collected, and after the intervention, the occurrence or absence of PsI was recorded and described.

Finally, at follow-up examinations at 6 and 12 months, the FMPI and FMBI were collected for all teeth, while the other previously recorded variables were evaluated only for the treated recessions. In addition, the CRC (the number of treated recessions with REC = 0 mm) was recorded, and the CRC% (CRC × 100/number of recessions) and the MRC% (mean preoperative REC-mean postoperative REC/mean preoperative REC × 100) were calculated.

The primary outcome was the percentage of mean root coverage (MRC%) and the secondary outcomes were the percentage of complete root coverage (CRC%), changes in the periodontal soft tissues variables, and the postsurgical incidences (PsI).

### Patient-reported outcome measures

In this study, the acute postoperative pain and the patient esthetic perception were evaluated. Each participant filled the UPV/EHU pain diary [16], where the duration and the intensity of their pain perception were measured in different established time points (including the first 24 h) using the visual analogue scale (VAS = 0–100). The pain diary was completed until the pain disappeared completely. Both the diary and the instructions to correctly fill it in were given (AFJ) to the patients on the day of the surgery.

Also, patients were asked about their satisfaction with the esthetic result, 6 and 12 months after the surgery. A VAS was used, with 0 indicating no esthetics and 100 the best possible esthetics.

### Sample size calculation

Using the MRC% as the primary outcome variable, it was estimated that with an SD = 24.86% [12], an *α*-risk of 5% and a statistical power of 80%, 22 patients would be needed in total. However, the sample was increased to 24 patients to compensate for possible dropouts.

### Statistical analysis

All the obtained data were analyzed using statistical software (IBM SPSS® Statistics 20.0;IBM, Chicago, IL, USA), with the patient as the unit of analysis. Initially, the Shapiro–Wilk test was used to evaluate if the distribution was normal or not. First, descriptive statistics were performed, and means and standard deviations were provided for quantitative variables, and percentages were determined for categorical variables. Subsequently, in the analysis of statistical relationships, normality tests led to the use of nonparametric tests for both intragroup (Wilcoxon test of the ranges for related samples) and intergroup comparisons (Mann–Whitney *U*). Finally, the possible relationship between the MRC% and the other variables at 6 and 12 months after the intervention was evaluated using the Spearman correlation coefficient, the Mann–Whitney *U* test or the Kruskal–Wallis test, according to the nature of the variable. The level of significance was set at *p* < 0.05.

## Results

### Study population and external validity

**Fig. 3:**
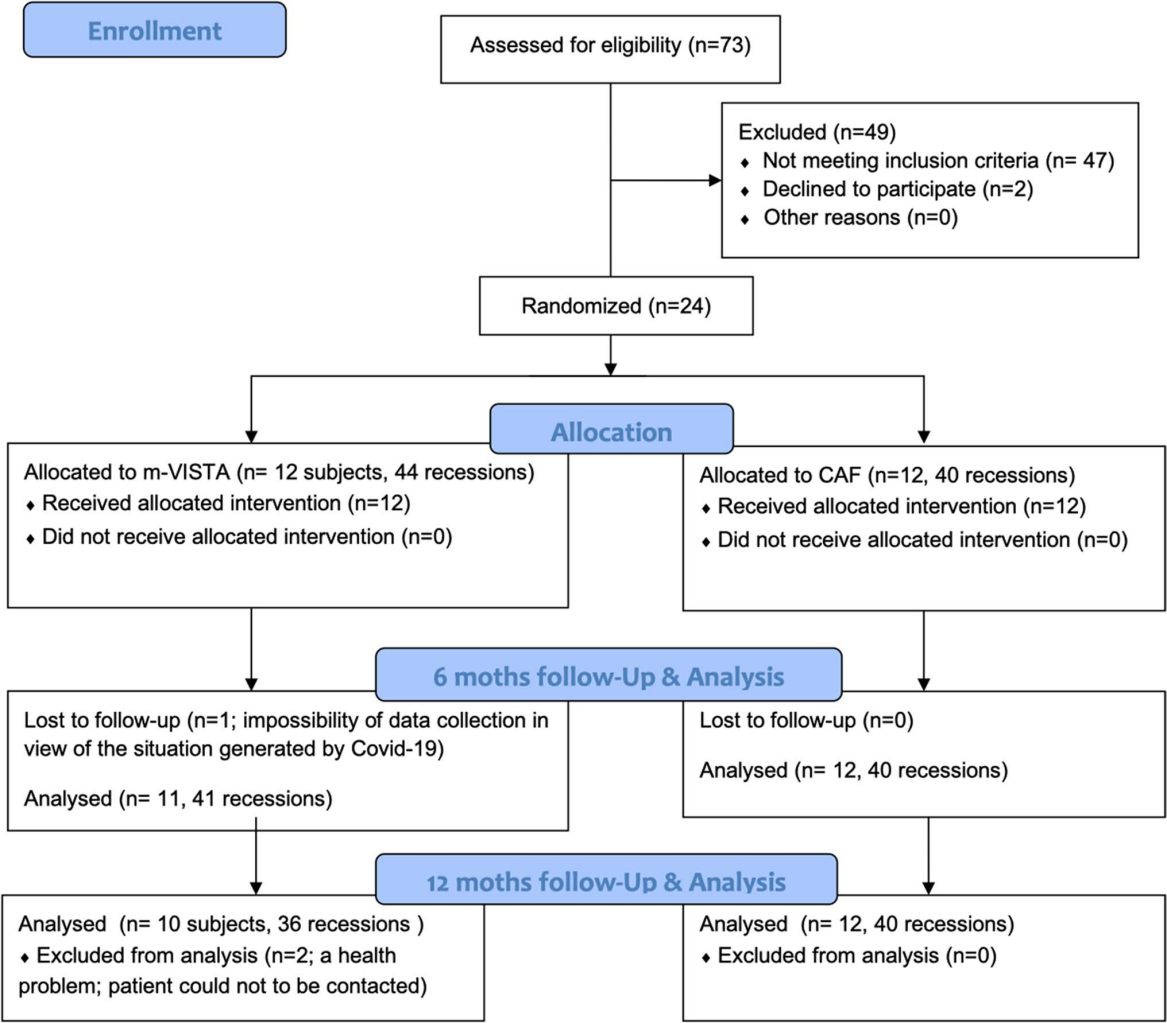
CONSORT flow diagram

The CONSORT diagram for patient recruitment is shown in Fig. 3. A total of 24 patients were included, 12 of which were assigned to the TG [8 women; 55.26 years (SD: 7.89)], and the other 12 were assigned to the CG [6 women; 51.16 years (SD: 10.37)]. During the follow-up period, two patients in the TG were lost.

The initial sociodemographic characteristics of the patients and the recessions are described in Tables 1 and 2, respectively. No statistically significant differences between the study groups were observed at baseline. Most of the treated recessions were located in the mandible (TG = 36 vs. CG = 29; *p* = 1.00), and in the TG, there was a greater number of recessions located in the posterior teeth (premolars: TG = 19 vs. CG = 16; molars: TG = 5 vs. CG = 3).

**Table 1:** Sociodemographic characteristics of the patients

Patients	Sex	Age (years)	Systemic disease	Medication	Smoking habits			Drugs/alcohol
					Type	cig/day	Years	
m-VISTA (*n* = 12)								
1	F	57	Fibromyalgia/migraine	Citalopram/lorazepam	FS	0	8	No
2	F	42	Hypercholesterolemia/arthrosis	No	NS	0	0	No
3	M	57	No	No	NS	0	0	No
4	M	60	Renal insufficiency/asthma/hypercholesterolemia	Terbutaline/rocatrol	FS	0	11	No
5	F	51	No	No	FS	0	32	No
6	F	52	No	No	NS	0	0	No
7	M	62	No	No	NS	0	0	No
8	F	55	Asthma/depression	Terbutaline/budesonide/formoterol/escitalopram	FS	0	11	No
9	F	47	No	No	NS	0	0	No
10	F	57	Anxiety/depression	Simvastatin/desvenlafaxine	FS	0	16	No
11	F	73	Depression	No	NS	0	0	No
12	M	51	No	No	FS	0	16	No
CAF (*n* = 12)								
1	F	50	Migraine	No	FS	0	6	No
2	F	59	No	No	FS	0	8	Yes
3	M	49	Asthma	Salbutamol	S	10	31	Yes
4	M	51	No	No	S	9	22	No
5	F	58	No	No	FS	0	12	Yes
6	M	56	Arterial hypertension	No	FS	0	27	No
7	F	31	No	Omeprazole	FS	0	6	No
8	M	63	No	No	NS	0	0	No
9	M	31	No	No	S	2	10	Yes
10	F	51	No	No	NS	0	0	No
11	M	59	No	No	NS	0	0	No
12	F	56	No	No	FS	0	36	No

*F*, female; *M*, male; *FS*, former smoker; *S*, smoker; *NS*, non-smoker; *cig/day*, cigarettes/day

**Table 2:** Characteristics of the treated Miller class III/RT2 multiple gingival recessions

Patients	FMPI (%)	FMBI (%)	*n*	Location		Recession										
				Arch	Tooth	PD (mm)	REC (mm)	CAL (mm)	GRW (mm)	KGW (mm)	CP-IP (mm)	CP-IP m (mm)	CP-IP d (mm)	RxBL (mm)	RxBL m (mm)	RxBL d (mm)
m-VISTA (*n* = 12)																
1	20.67	6	4	Md	3.1,3.2,3.4,3.5	1.25	3.25	4.50	3.25	2	2.50	1	3	4.90	4.76	5.04
2	11.90	17.26	5	Md	4.1,4.2,4.4,4.5,4.6	1.20	2.40	3.60	3.80	2.80	3.10	3	3.20	4.41	4.71	4.11
3	4.17	3.47	3	Md	3.4,3.5,3.6	2	4.33	6.33	6.33	2.67	3	3.33	2.67	3.48	3.57	3.39
4	13.89	9.72	4	Mx	2.1,2.2,2.3,2.5	1	2.50	3.50	2.75	3	3.43	3.33	3.50	3.08	2.98	3.18
5	24.36	13.46	4	Mx	2.1,2.2,2.3,2.4	2	2.50	4.50	3.25	4.75	4.33	4.33	4.33	3.37	3.76	2.98
6	6.79	4.32	3	Md	4.4,4.5,4.6	1.67	2.33	4	5.33	4.33	0.33	0.33	0.33	4.93	5.23	4.64
7	12.18	6.41	4	Md	4.3,4.4,4.5,4.6	2	2.75	4.75	4.50	1	0.50	0.25	0.75	2.51	2.47	2.54
8	6	6	4	Md	4.3,4.4,4.5,4.6	2.25	3.25	5.50	5.25	2.50	2.25	1.75	2.75	7.49	7.83	7.15
9	6.48	4.63	4	Md	3.2,3.3,3.4,3.5	2	2.50	4.50	3.50	1.50	0.88	1	0.75	2.44	2.40	2.48
10	11.40	7.89	3	Md	3.2,3.3,4.3	2.33	4	6.33	3.67	2.33	2	2.67	1.33	5.79	6.22	5.36
11	20	30	3	Md	3.2,3.3,3.4	1.33	2	3.33	3.67	2.67	2.83	3	2.67	4.69	4.66	4.71
12	12.67	10.67	3	Md	3.3,3.4,3.5	2	2.33	4.33	3.67	3	0	0	0	3.11	3.42	2.80
Mean (SD)	12.54 (6.38)	9.99 (7.50)	44	10 Md; 2 Mx	5 Ms; 19 PMs; 9 Cs; 11 Is	1.75 (0.45)	2.85 (0.72)	4.60 (1.01)	4.08 (1.06)	2.71 (1.05)	2.10 (1.38)	2 (1.46)	2.11 (1.41)	4.18 (1.49)	4.34 (1.59)	4.03 (1.41)
CAF (*n* = 12)																
1	15.53	11.11	3	Md	3.1,3.2,3.3	2.67	5.67	8.33	3.67	1	6.33	6.67	6	5.79	5.12	6.46
2	8.33	4.22	3	Md	4.4,4.5,4.6	1.67	2.67	4.33	4.33	2.33	0.50	0.67	0.33	2.65	2.70	2.60
3	13.33	14	3	Mx	1.3,1.4,1.5	1.33	3.33	4.67	4.33	1.67	3.50	3.67	3.33	3.98	4.16	3.80
4	19.57	11.59	3	Md	4.2,4.3,4.4	2	2.67	4.67	4	1.67	2.50	2.50	2.50	4	4.01	3.99
5	7.14	11.11	3	Md	3.1,3.3,4.3	1	2.67	3.67	2.33	3	1.83	1.67	2	5.78	5.66	5.90
6	23.08	7.69	3	Md	3.3,3.4,3.5	1.67	2.33	4	3.33	3.33	1.83	2.33	1.33	4.39	4.33	4.46
7	5.36	6.55	3	Md	4.4,4.5,4.6	2	2.67	4.67	2	2.33	2.33	2.33	2.33	3.10	3.10	3.11
8	10.67	10	3	Mx	1.3,1.4,1.5	2	2.33	4.33	3.33	5	1.50	2	1	4,04	4.42	3.65
9	9.88	1.85	3	Md	4.1,4.3,4.4	1.33	4.33	5.67	4	1.67	4.60	4.67	4.50	4.04	3.91	5.04
10	8.33	6.94	4	Md	4.3,4.4,4.5,4.6	2.50	4.50	7	3.75	1.50	3.13	2.50	3.75	3.14	2.92	3.36
11	22.50	19.17	4	Md	3.2,3.3,4.2,4.3	2.75	3.50	6.25	3	2.75	1.50	1.50	1.50	4.76	4.81	4.71
12	18.45	8.93	5	Mx	1.1,1.2,1.3,1.4,1.5	1.80	3.20	5	4.80	3.80	2.10	2.20	2	3.30	3.44	3.16
Mean (SD)	13.51 (6.17)	9.43 (5.46)	40	9 Md; 3 Mx	3 Ms;16 PMs; 12 Cs; 9 Is	1.89 (0.55)	3.32 (1.03)	5.22 (1.37)	3,57 (0.83)	2.50 (1.14)	2.64 (1.58)	2.73 (1.60)	2.55 (1.60)	4.12 (1.00)	4.05 (0.90)	4.19 (1.17)

*FMPI*, full mouth plaque index; *FMBI*, full mouth bleeding index; *n*; number; *Md*, mandible; *Mx*, maxilla; *Ms*, molars; *PMs*, premolars; *Cs*, canines; *Is*, incisors; *PD*, probing depth; REC, recession; *CAL*, clinical attachment level; *GRW*, gingival recession width; *KGW*, keratinized gingiva width; *CP-IP*, distance from the contact point to the interdental papilla; *m*, mesial; *d*, distal, *RxBL*; radiological bone level; *SD*, standard deviation

### Clinical results at 6 and 12 months of follow-up

The clinical results are showed in Tables 3 and 4. Gingival recessions were significantly reduced at 6 and 12 months in both groups (TG: *p* < 0.001 (6 and 12 months)/CG: *p* < 0.002 (6 months) and *p* < 0.02 (12 months)), although there were no statistically significant differences between the groups (Table 4).

**Table 3:** Clinical results at 6 and 12 months after the corresponding periodontal plastic surgery

Patients	FMPI (%)	FMBI (%)	PD (mm)	REC (mm)	MRC		CRC			CAL (mm)	GRW (mm)	KGW (mm)	CP-IP (mm)	CP-IP m (mm)	CP-IP d (mm)
					mm	%	*n*	%	Loc						
6 months															
m-VISTA (*n* = 11)															
Mean (SD)	14.67 (6.47)	8.96 (5.04)	1.75 (0.43)	1.12 (0.74)	1.90 (0.78)	61.59 (25.24)	1.18 (1.4) (*n* = 13)	30.61 (36.46)	1 Ms; 4 PMs; 4Cs; 4 Is	2.87 (0.76)	1.94 (1.27)	3.57 (1.62)	1.23 (0.98)	1.06 (0.97)	1.32 (1.06)
CAF (*n* = 12)															
Mean (SD)	24.58 (23.71)	13.46 (10.55)	1.97 (0.51)	1.39 (1.25)	1.93 (0.92)	61.73 (30.29)	1.17 (1.19) (*n* = 14)	36.67 (38.82)	2 Ms; 4 PMs; 6 Cs; 2 Is	3.37 (1.55)	2.56 (1.66)	3.18 (0.88)	2.27 (1.64)	2.23 (1.71)	2.31 (1.57)
Intergroup *p*	0.41	0.49	0.21	0.93	0.57	** > **0.05	0.93	0.74	**-**	0.61	0.29	0.61	0.08	0.05	0.13
12 months															
m-VISTA (*n* = 10)															
Mean (SD)	15.86 (9.50)	8.83 (4.85)	1.73 (0.57)	0.87 (0.82)	2.11 (0.63)	73.26 (23.64)	1.7 (1.63) (*n* = 17)	46.67 (44.31)	2 M; 9 PMs; 3 Cs; 3 Is	2.43 (1.14)	1.37 (1.26)	3.58 (1.66)	1.84 (1.41)	1.87 (1.53)	1.77 (1.40)
CAF (*n* = 12)															
Mean (SD)	20.49 (14.07)	12.71 (14.72)	1.93 (0.54)	1.54 (1.16)	1.78 (0.92)	56.49 (29.27)	0.92 (1.08) (*n* = 11)	29.45 (36.23)	1 M; 4 PMS; 4 Cs; 2 IS	3.47 (1.08)	2.32 (1.45)	3.08 (1.12)	2.31 (1.35)	2.43 (1.42)	2.19 (1.31)
Intergroup *p*	0.54	0.92	0.31	0.16	0.25	0.20	0.31	0.46	**-**	**0.04**	0.16	0.58	0.31	0.42	0.38

*FMPI*, full mouth plaque index; *FMBI*, full mouth bleeding index; *PD*; probing depth; *REC*, recession; *MRC*, mean root coverage; *CRC*, complete root coverage; *n*, number; *Loc*, localization; *Ms*, molars; *PMs*, premolars; *Cs*, canines; *Is*, incisors; *CAL*, clinical attachment level; *GRW*, gingival recession width; *KGW*, keratinized gingiva width; *CP*-IP, distance from the contact point to the interdental papilla; *m*, mesial; *d*, distal; *SD*, standard deviation

**Table 4:** Results of clinical changes achieved after the corresponding periodontal plastic surgery

	∆ basal–6 months					∆ basal–1 year					∆ 6 months–1 year				
	m-VISTA (*n* = 11)		CAF (*n* = 12)		*p*	m-VISTA (*n* = 10)		CAF (*n* = 12)		*p*	m-VISTA (*n* = 10)		CAF (*n* = 12)		*p*
	Mean (SD)	*p*	Mean (SD)	*p*		Mean (SD)	*p*	Mean (SD)	*p*		Mean (SD)	*p*	Mean (SD)	*p*	
MRC (mm)	1.8 (0.81)	**0.01**	1.93 (0.92)	**0.002**	0.57	2.11 (0.01)	**0.01**	1.78 (0.92)	**0.02**	0.25	0.21 (0.50)	0.32	− 0.15 (0.63)	0.57	0.25
MRC (%)	61.59 (25.24)	-	61.73 (30.29)	-	> 0.05	73.26 (23.64)	-	56.49 (29.27)	-	0.20	8.85 (20.48)	0.23	− 5.23 (19.90)	0.40	0.12
PD (mm)	− 0.04 (0.51)	0.81	0.08 (0.75)	0.68	0.79	− 0.12 (0.84)	0.34	0.04 (0.65)	0.73	0.25	0.05 (0.80)	0.59	− 0.04 (0.74)	0.84	0.92
CAL (mm)	− 1.84 (1.07)	**0.003**	− 1.85 (1.28)	**0.005**	0.79	− 2.40 (1.43)	**0.01**	− 1.74 (1.16)	**0.003**	0.18	− 0.43 (1.24)	0.26	0.11 (0.92)	0.61	0.16
GRW (mm)	− 2.17 (1.25)	**0.003**	-1.01 (1.40)	0.06	0.08	− 2.78 (1.78)	**0.01**	− 1.26 (1.07)	**0.007**	0.06	− 0.49 (1.03)	0.09	− 0.24 (1.26)	0.20	0.82
KGW (mm)	0.85 (1.19)	0.06	0.68 (0.87)	**0.02**	0.65	0.88 (0.84)	**0.02**	0.58 (1.10)	0.09	0.35	0.11 (1.29)	0.89	− 0.10 (1.25)	0.45	0.82
CP-IP (mm)	− 0.80 (0.91)	**0.02**	− 0.37 (1.93)	0.59	0.38	− 0.08 (0.90)	0.72	− 0.33 (2.23)	0.89	0.77	0.68 (0.83)	**0.01**	0.04 (1.58)	0.88	0.14
CP-IP m (mm)	− 0.85 (1.02)	**0.02**	− 0.49 (1.94)	0.41	0.57	− 0.07 (0.80)	0.73	− 0.29 (2.27)	0.97	0.97	0.84 (0.90)	**0.01**	0.20 (1.64)	0.93	0.20
CP-IP d (mm)	** − **0.73 (0.87)	**0.02**	** − **0.24 (1.95)	0.72	0.32	** − **0.18 (1.01)	0.44	** − **0.36 (2.24)	0.81	0.77	0.55 (0.80)	0.08	− 0.12 (1.53)	0.56	0.12

*∆*, change; *MRC*, mean root coverage; *n*, number; *PD*, probing depth; *CAL*, clinical attachment level; *GRW*, gingival recession width; *KGW*, keratinized gingiva width; *CP-IP*, distance from the contact point to the interdental papilla; *m*, mesial; *d*; distal; *SD*, standard deviation

At 6 months, the MRC% was similar for both groups [TG = 61.59% (95% *CI*; 44.64–78.54) vs. CG = 61.73% (95% *CI*; 42.48–80.97); *p* ≥ 0.05], while at 12 months, the MRC% increased to 73.26% (95% *CI*; 56.36–90.17) in the TG and decreased to 56.49% (95% *CI*: 37.90–75.10) in the CG, this difference not being statistically significant between the two groups (*p* = 0.20) (Table 3).

In addition, in the TG, a negative linear correlation was observed between the MRC% at 6 months and the RxBL (mean: *r* =  − 0.68, *p* = 0.02; mesial: *r* =  − 0.71, *p* = 0.014; distal: *r* =  − 0.67, *p* = 0.03) and between the MRC% at 12 months and the PC-IP at baseline (mean: *r* =  − 0.702, *p* = 0.02; mesial: *r* =  − 0.65, *p* = 0.04; distal: *r* =  − 0.74, *p* = 0.01) and the RxBL on the distal surface (*r* =  − 0.64; *p* = 0.05). On the other hand, in the CG, a positive linear correlation was detected between the MRC% and the width of the CTG at both 6 months (*r* = 0.65; *p* = 0.02) and 12 months (*r* = 0.59; *p* = 0.04), and a negative linear correlation was observed between the MRC% and the FMPI (*r* =  − 0.81; *p* = 0.001) and the FMBI (*r* =  − 0.64; *p* = 0.02) at 6 months.

Respecting CRC%, it was calculated at patient and recession levels, 6 and 12 months after the intervention. At 6 months, at least one recession with CRC was observed in 54.54% (*n* = 6/11) of the TG patients and in 58.33% (*n* = 7/12) of the CG patients; furthermore, CRC was observed for all treated recessions in one patient in the TG and two patients in the CG. At recession level, the CRC% was of 31.71% (*n* = 13/41) in the TG and 36.67% (*n* = 14/40) in the CG.

At 12 months, the CRC% increased in the TG at both, the patient level (all recessions were covered in three patients, and at least one recession was covered in 60% of patients) and the recession level (47.33%; *n* = 17/36). In contrast, in the CG, the CRC% decreased at both levels, with all recessions covered in one patient and at least one recession covered in 50% of the patients; this resulted in a CRC of 29.45% (*n* = 11/40). There were no statistically significant differences between the two study groups at either the patient or the recession levels at any follow-up point (Table 3).

The CAL data are reported in Tables 3 and 4. At 12 months, the CAL decreased to 2.43 mm in the TG and increased to 3.47 mm in the CG, being the only statistically significant difference (*p* = 0.04) found between both treatment groups.

On the other hand, the reductions in GRW at 6 months [TG =  − 2.17 mm vs. CG =  − 1.01 mm; *p* = 0.08] and at one year [TG =  − 2.78 mm vs. CG =  − 1.26 mm; *p* = 0.06] compared to the initial values were higher in the TG group, while the KGW gain was < 1 mm in both groups at 6 months (TG = 0.85 mm vs. CG = 0.68 mm; *p* = 0.65), with minimal changes at 1 year and no statistically significant differences between the groups (Table 4).

Regarding the increase in the papilla, the TG showed a significant reduction in the PC-IP distance at 6 months, both mesially (TG =  − 0.85 mm vs. CG =  − 0.49 mm; *p* = 0.02) and distally (TG =  − 0.73 mm vs. CG =  − 0.24 mm; *p* = 0.02), compared to baseline. However, at 12 months, a regression of these values was observed, and this change was statistically significant (*p* = 0.01) between 6 and 12 months. Nonetheless, no statistically significant differences were observed between the groups at any follow-up point (Table 4).

Finally, in the TG, the FMPI was 14.67% and the FMBI was 8.96% at 6 months and, at 1 year, the FMPI was 15.86% and the FMBI was 8.83%. However, in the CG, these values were higher at both 6 months and one year, with FMPIs of 24.58% and 20.49% and FMBIs of 13.46% and 12.71%, respectively (Table 3).

### Intraoperative and postsurgical results

Regarding the dimensions of the CTGs, their lengths (TG = 28.88 mm vs. CG = 26.35 mm) and widths (TG = 7.44 mm vs. CG = 6.95 mm) were greater in the TG. In contrast, the thickness (TG = 2.36 mm vs. CG = 2.61 mm) was greater in the CG. There were no statistically significant differences between the two groups for any of the CTG characteristics.

A total of 12 PsI were recorded, including facial haematoma (TG = 1 vs. CG = 2), aphthae (TG = 2 vs. CG = 1), necrosis of the palate (TG = 2 vs. CG = 2), partial necrosis of the flap (TG = 0 vs. CG = 1), partial necrosis of the graft (TG = 0 vs. CG = 1), and postsurgical bleeding (TG = 1 vs. CG = 0).

### Patient-reported outcomes

Both mean pain intensity (PI) (TG = 11.19 vs. CG = 8.10) and pain duration (PD) (TG = 25.27 min vs. CG = 10.34 min) were higher after the intervention in the TG, although they were only statistically significant at 2 (PI and PD: p = 0.001) and 8 h (PI: *p* = 0.045 / PD: *p* = 0.010) after the intervention.

The level of satisfaction with the esthetic result was high, both at 6 (TG = 82.18 vs. CG = 78.33) and 12 months (TG = 83.80 vs. CG = 80.75) of follow-up. No statistically significant differences were found between the groups.

## Discussion

In this clinical study, the MRC% achieved by means of two different surgical procedures was compared. Considering the evidence [5–7], this would be the first RCT assessing the efficiency in terms of RC of two techniques in multiple Miller class III [3]/RT2 [4] gingival recessions. The MRC% obtained was of nearly 61% in the m-VISTA technique [16] group (TG) and in the CAF technique [14] group (CG) at 6 months. Nevertheless, in the TG, the MRC% increased to 73.26% and in the CG decreased to 56.49% at 12 months, without statistically significant differences between the groups. To date, few RCTs have reported the treatment of Miller class III [3]/RT2 [4] gingival recessions [[5–7], most with a 6-month follow-up [8–11, 27–31]. Only three studies had a follow-up period of at least 1 year [10, 12, 13], as in the present study. When MRC% was assessed at 6 months [8–11, 27–31], it ranged from 56.68 to 95.10%, which would be in concordance with our results, while the range at 12 months [62.83–79.10%], reported only by three authors [10, 12, 13], would only include the results described for the TG. However, it should be noted that the literature ranges are very wide and this might be explained by the surgical technique [32] or the so-called center effect [33, 34], among other factors.

On the one hand, the increase in the MRC% in the TG from 61.59 to 73.26% at 12 months, despite presenting some more unfavorable baseline characteristics could be related to the traction and coronal stabilization of the tunnel-papillae-graft complex, which would favor the maturation of marginal soft tissues over time. On the other hand, the reduction in the MRC% in the CG could be due to the characteristics of the patients, who showed a tendency to higher FMPI and FMBI values and greater tobacco consumption [35]. Additionally, this group experienced some PsI in the recipient site (partial necrosis of the graft and flap in two patients, respectively), which could have impaired the RC results.

Also, different characteristics of the recessions can facilitate or hinder the RC obtained in Miller class III/RT2 recessions, including the tooth position [36, 37], the width and height of the recession [7, 38], and the integrity of the interdental papilla [12, 39].

In fact, when comparing all the recessions included in this study with those treated in recent RCTs with a 12-month follow-up [12, 13], some differences could be observed regarding the location of the recessions. In the present study, 77.38% of the gingival recessions were located in the mandible, with 51.19% of them being in the posterior area, including 8 molars; while in the study of Mercado et al. [13], only antero-inferior teeth were treated. Finally, Aroca et al. [12] also included five molars and 50% of the lesions were located in the mandible.

In addition, our recessions were smaller (3.10 mm) than those treated by Mercado et al. [13] (5.61 mm) and, additionally, their KGW was lower (1.74 mm compared to 2.60 mm in this study), possibly due to the location. In contrast, the recessions treated in the present study were quite similar to those treated by Aroca et al. [12] in terms of size, GRW, and CP-IP. Therefore, according to the literature, the characteristics of the recessions in this study would be more unfavorable in terms of location (mandibular [37, 40] and posterior teeth [36, 41]) and size, since, the greater the depth [7, 42] and initial width of the recession, the larger the avascular area of the surface to be treated [29]. This could partly explain the lower total MRC% at 12 months in our study (64.86%, compared to 75.25% [13] and 79.10% [12], respectively).

As previously mentioned, when comparing the MRC% of the study groups independently, the results favored the TG, although in this group, there was a greater number of mandibular and posterior recessions. This could be due to the positive influence of other baseline characteristics, such as a smaller recession size, greater soft tissue filling in the interproximal space, and greater KGW.

In fact, some of these clinical variables could have a stronger or weaker influence on the MRC% according to the periodontal plastic technique used. Thus, with the CAF technique, a positive association was observed between the MRC% and the width of the CTG, and a negative relationship was observed between the MRC% and the FMPI and FMBI, while with the m-VISTA technique, there was a negative association between the MRC% and the RxBL and PC-IP. The latter association is consistent with the results of Aroca et al. [43], who obtained a higher RC for this type of multiple recessions when they were located in the maxilla and when there was a greater presence of papilla in the interproximal areas of the upper jaw.

This study highlights the difficulty of achieving CRC% it in this type of recessions, with 46.7% of the recessions in the TG and 29.5% in the CG being completely covered at 12 months. This corroborates the findings of a previous systematic review that reported values of 42.1% at 12 months, with a decrease to 18.23% when the follow-up was extended beyond 1 year [5]. Therefore, when assessing the success of this treatment, it may be more reasonable to record the MRC% along with other clinical parameters, including KGW and PC-IP.

Thus, in this study at 12 months, the KGW gain was 0.88 mm in the TG and 0.58 mm in the CG, which is within the ranges [0.10–1.72 mm] previously described [10, 12, 13]. In addition, regarding the PC-IP, Aroca et al. [12], who performed a tunnel with coronal traction, showed a reduction of 1.15 mm at 6 months that remained stable at 1 year of follow-up. In the present study, this reduction was less than 1 mm at 6 months in both groups, although the change was statistically significant in the TG, probably due to the coronal mobilization achieved by the m-VISTA technique. Nonetheless, this reduction did not remain stable at 1 year and returned almost to the initial values. This could be a consequence of the maturation of the tissues, as the position of the interdental papilla depends on the distance between the contact point and the underlying alveolar bone crest [44]. Thus, the CTG would be unable to support the postsurgical position of the papilla, contrary to the hypothesis described by Aroca et al. [43].

While patients were highly satisfied with the esthetic results, postoperative pain was of greater intensity and duration in patients who underwent surgery with the m-VISTA technique. Likewise, Gobbato et al. [45] reported a greater incidence of pain in the tunnelling technique compared to the CAF, both combined with a CTG. Although these variables are very important nowadays, caution should be taken when assessing pain, as the data should be analyzed associating them with an adequate recording of the predisposing or perpetuating factors of pain present in the subjects of the study.

Finally, this RCT is not exempt from limitations, such as not having considered the thickness of the flap, as the current classification was not available when this study was designed, the participation of postgraduate students in surgical procedures, and the follow-up (12 months), which did not allow the evaluation of the long-term stability of the results. There is no doubt that these aspects should be analyzed in future studies.

However, it is the authors’ belief that this study has its strengths, such as the significantly good results in terms of REC reduction in multiple Miller class III [3]/RT2 [4] recessions (at least 3 recessions located in the same quadrant), as well as the evaluation of the patient’s perception and the acute postsurgical pain within the first 24 h after the surgery.

## Conclusion

Within the limitations of this study, it could be concluded that there were no statistically significant differences regarding the MRC% achieved in the treatment of multiple Miller class III/RT2 recessions with both surgical techniques. In addition, when success was assessed considering not only CRC% but also other periodontal clinical parameters, the results favored treatment with the m-VISTA technique. Finally, more RCTs with larger samples and longer follow-ups are needed to compare the use of different periodontal plastic techniques for the treatment of multiple Miller class III/RT2 recessions and thus define the technique of choice for each case.

## Supplementary Information

Below is the link to the electronic supplementary material.

Supplementary file1 (PDF 4512 KB)

## Data Availability

The data that supported the findings of this study are available from the corresponding author upon reasonable request.
